# Partial Discharge Characteristics of Polymer Nanocomposite Materials in Electrical Insulation: A Review of Sample Preparation Techniques, Analysis Methods, Potential Applications, and Future Trends

**DOI:** 10.1155/2014/735070

**Published:** 2014-01-16

**Authors:** Wan Akmal Izzati, Yanuar Z. Arief, Zuraimy Adzis, Mohd Shafanizam

**Affiliations:** Institute of High Voltage and High Current, Faculty of Electrical Engineering, Universiti Teknologi Malaysia (UTM), 81310 Johor Bahru, Johor, Malaysia

## Abstract

Polymer nanocomposites have recently been attracting attention among researchers in electrical insulating applications from energy storage to power delivery. However, partial discharge has always been a predecessor to major faults and problems in this field. In addition, there is a lot more to explore, as neither the partial discharge characteristic in nanocomposites nor their electrical properties are clearly understood. By adding a small amount of weight percentage (wt%) of nanofillers, the physical, mechanical, and electrical properties of polymers can be greatly enhanced. For instance, nanofillers in nanocomposites such as silica (SiO_2_), alumina (Al_2_O_3_) and titania (TiO_2_) play a big role in providing a good approach to increasing the dielectric breakdown strength and partial discharge resistance of nanocomposites. Such polymer nanocomposites will be reviewed thoroughly in this paper, with the different experimental and analytical techniques used in previous studies. This paper also provides an academic review about partial discharge in polymer nanocomposites used as electrical insulating material from previous research, covering aspects of preparation, characteristics of the nanocomposite based on experimental works, application in power systems, methods and techniques of experiment and analysis, and future trends.

## 1. Introduction

Polymers are widely used as insulation material in high voltage systems due to their high breakdown strength under electrical stress. Previously, the conventional polymer microcomposite with added filler such as silica (SiO_2_), alumina (Al_2_O_3_), and titania (TiO_2_) has been developed, as it may produce better properties than polymer alone. In electrical systems, partial discharges (PD) have always been a predecessor to major faults in electrical insulation such as glass, ceramic, polymers, and composite material. The occurrence of PD may alter the dielectric properties of these materials, making them less effective as insulators.

For this reason, researchers in the last decade have developed a new material, polymer nanocomposite (also known as nanofiller-added polymers), which may replace conventional polymer composites with enhanced properties [1–12]. The new material has overcome the drawbacks of polymer composite materials, thus providing significant improvement in terms of mechanical and electrical erosion reduction, mechanical strength enhancement, electrical breakdown/endurance behavior, and space charge mitigation. Many studies have proven that polymer nanocomposite has better PD characteristics evaluated by erosion depth, amplitude of PD, and surface morphology of polymer nanocomposite specimens. The study of PD has become a tool in assessing the quality and performance characteristics of high voltage equipment.

This paper provides a comprehensive review of partial discharge on polymer nanocomposites in the field of high voltage insulation. We discuss the concept of nanocomposites, the role of nanoparticles in polymer nanocomposites, sample preparation, experimental work and findings, applications in power systems, methods and techniques involved in the experimental work from previous studies regarding the partial discharge characteristics of various types of nanocomposites, and future trends and challenges in this field.

## 2. Information and Analysis

### 2.1. Concept of Nanocomposites

Polymer nanocomposites are defined as composites in which small amounts of nanometer sized fillers are homogeneously distributed in a polymer by certain weight percentages (wt%). Polymers such as polyethylene (PE), polypropylene (PP), polyamide (PA), epoxy, and rubber are combined with nanofillers that can be either alumina (Al_2_O_3_), titania (TiO_2_), silica (SiO_2_), magnesium oxide (MgO), clay, or other new materials proposed. For a better understanding of nanocomposites, the different structures and dimensions of nanocomposites and conventional microcomposites should be compared, clarified, explained, and elaborated clearly. According to Tanaka et al. [13], comparison between nanocomposite and microcomposite polymer materials can be done based on three major properties: content of fillers, size of fillers, and specific surface area of fillers. The comparison is seen in Table 1 [13].

Nanocomposites require a smaller amount of fillers than microcomposites. Therefore, polymer nanocomposites are almost pure polymer, such that some properties of the polymer remain unaffected even after becoming polymer nanocomposites, such as the density of the composites. Besides, with smaller amounts of fillers, the distance between neighboring filler in nanocomposites will be smaller than in conventional microcomposites. Lastly, nanocomposites have a specific surface area that is three orders larger than microcomposites [13]. Thus, interaction of polymer matrices with fillers is expected to be much greater in nanocomposites. These properties may have a further impact on the behavior of insulation in electrical systems. We may expect improvements in electrical properties such as higher breakdown strength, higher resistance to partial discharges, and treeing, as well as in their mechanical and chemical properties. With the rapid development of nanotechnology research, the fabrication of insulation can be improved.

**Figure 1:**
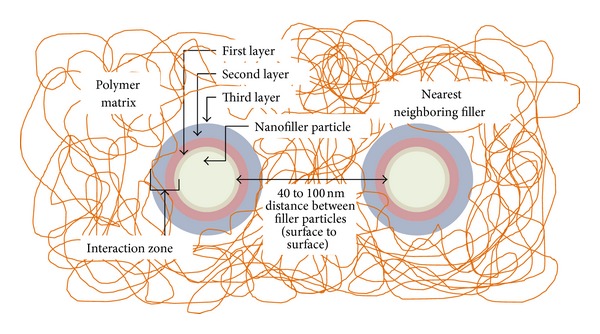
Main constituents inside a polymer nanocomposite composed of polymer matrix, nanofillers, and interaction zone [15].

Basically, polymer nanocomposites have three major constituents: polymer matrix, nanofillers, and interaction zone.  Figure 1 shows a simplified illustration of the constituents of polymer nanocomposites [14]. The constituents are discussed further as follows.

*(a) Polymer Matrix.* The polymer matrix has three main categories: thermoplastics, thermosets, and elastomers. Thermoplastic is from the word “thermo” which means “heat” and “plastic” is “polymer.” Thermoplastic material can be softened when heated and can become softer as the heat increases. Thermosets are materials that are heated and vulcanized to produce a cross-linked structure that ties the polymer chains to a given shape, while elastomers are elastic materials that can deform when force is applied and revert to the original shape when the force is released, such as rubber.

*(b) Nanofillers.* Nanofillers are classified by their dimensions: one-dimensional—normally referred to as thin platelets, two-dimensional—referred to as nanowires or nanotubes, and three-dimensional—referred to as inorganic oxides [15]. Among researchers in the electrical field, the most popular nanofillers for insulation purposes are one-dimensional, such as clay or layered silicate, and three-dimensional, for example, alumina (Al_2_O_3_), silica (SiO_2_), titania (TiO_2_), and many more. 

*(c) Interaction Zone.* The interaction zone is the interfacial area between the matrix and the filler. According to Tanaka et al. [16], the interaction zones consist of three layers: bonded layer (first layer), bound layer (second layer), and loose layer (third layer). The bonded layer is a transition layer, having a thickness of 1 nm, which tightly bonds both inorganic and organic substances by coupling agents. The bound layer is a several nm layer of polymer chains that are strongly bound, interacting with the first layer (bonded layer) and inorganic particles. The loose layer loosely interacts with the second layer and has a thickness of several tens of nm. Above these three layers, there is an electric double layer which has a coulombic interaction that charges the nanoparticle positively or negatively.

### 2.2. Types of Polymer Nanocomposites

#### 2.2.1. Polymer/Layered Silicate Nanocomposites

**Figure 2:**
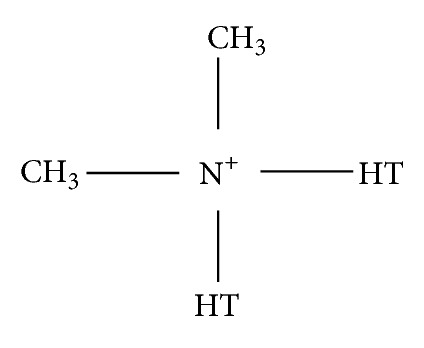
Molecular structure of Cloisite 15A [17].

Polymer/layered silicate nanocomposites are made with one-dimensional nanofillers. An example of a layered silicate that has been investigated is Cloisite 15A (natural montmorillonite clay), by Guastavino et al. [17]. This clay is modified with dimethyl ditallow ammonium and the molecular structure is shown in Figure 2. In the experiment, the combination of LDPE polymers and this layered silicate nanofiller results in improvements in terms of breakdown strength and space charge.

#### 2.2.2. Polymer/Metal Oxide Nanocomposites

Metal oxide filler is a three-dimensional nanofiller commonly used among researchers for insulation improvement in terms of dielectric properties. As an example of the metal oxides that have been investigated, Maity et al. [18] made use of alumina (Al_2_O_3_) and titania (TiO_2_) in their studies. In their experiment, the polymer matrix was epoxy resin (Bisphenol-A) and was combined with both alumina and titania. As a result, the surface degradation of nanocomposite-filled epoxy samples showed improvements compared with a neat epoxy resin sample and a microcomposite-filled epoxy resin sample.

### 2.3. Role of Nanoparticles in Polymer Nanocomposites

Many studies have reported nanocomposite filler giving better results in terms of electrical properties than microcomposite filler when used with polymer. These findings were confirmed by researchers in the high voltage insulation field. But the question is what is actually happening in these composites? How does the size of particles influence their properties? Is there any part of polymer nanocomposite that has the major role in this property enhancement?

The interaction zone or interfacial area is the main factor contributing to the improvement of the insulating properties of the nanocomposites. This is the area that interfaces between the polymer matrix and the nanofillers. Its role in property enhancement lies on the interaction zone due to its characteristic of having a specific surface area that is three orders larger than conventional microcomposite filler. This provides smaller distances between neighboring fillers [15]. Nanoparticles have a high surface area-to-volume ratio, which means that for the same particle loading, a nanocomposite will have a much greater interfacial area than microcomposite [15, 19]. Since the interaction zone for nanocomposite is far larger than for microcomposite, it has a great influence on the property improvement [19, 20].

The role of nanoparticles has been proven experimentally by Maity et al. [18], who found that nanoparticles bonded with the polymer matrix (epoxy resin) can resist surface erosion. Nanoparticles provide a superior interface region between polymer matrices, and thus a large volume of polymer belonging to the interfacial zone results in higher resistance against erosion. Normally, the degradation occurs in small isolated regions that form channels around existing nanoparticles [18], so good dispersion of nanoparticles will improve the resistance to degradation or erosion on the surface of the nanocomposite material.

**Figure 3:**
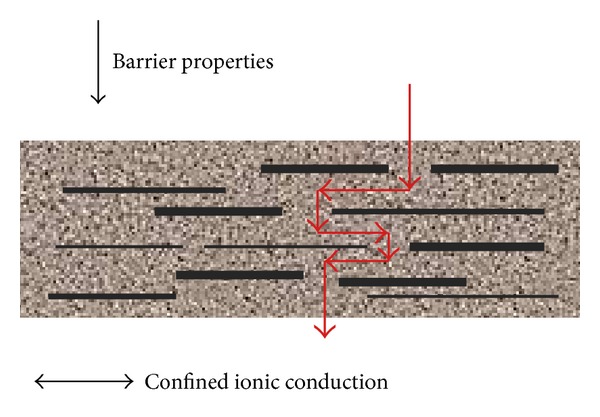
The barrier behavior of the nanoparticles inside polymer matrix [13].

With nanosize particles, it is possible to reinforce the polymer matrix and to improve the barrier resistance against gas and liquid permeation [13]. Cao et al. [21] also claimed that the nanoparticles alter the polymer structure to have a barrier behavior between their layered structure and the adjustable anisotropic ionic conductivity between the layers, as shown in Figure 3.

### 2.4. Polymer Nanocomposite Structures

**Figure 4:**
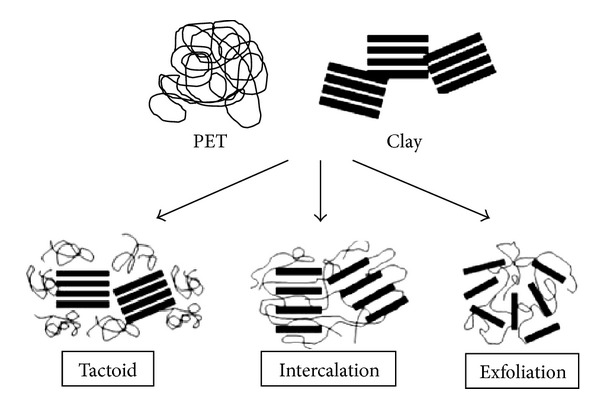
Three types of nanocomposite structures: (a) tactoid, (b) intercalation, and (c) exfoliation structure [22].

Previous fabrications of nanoparticles were difficult to disperse. Thanks to advanced developments in the processing technology of polymer nanocomposites, the nanoparticles are now easier to disperse more evenly. Modern nanocomposites are formed through shear intercalation and exfoliation, as demonstrated by the effective diffusion of polymer in between organophilic nanoparticles. Intercalation results in a well-ordered stacked multilayer structure of nanocomposites, which means there is a firm interfacial bond between the polymer and the nanocomposite. The exfoliation structure of the nanocomposite is well separated into single layers within a continuous polymer matrix. The bonding for intercalated or exfoliated layered nanocomposites is through a compatibilizer chemical added to the polymer matrix. Some nanocomposites may be formed in tactoid structures, which are structures of conventional composite, for cost reduction, but the nanocomposite plays a small role in property improvement. Thus, for the greatest dispersion and interfacial interaction between nanocomposites and polymer, the exfoliation structure is suggested [13, 22, 23].  Figure 4 shows an example of the three types of nanocomposite structures using clay when combined with polymer polyethylene (PET).

### 2.5. Partial Discharge Characteristics of Nanocomposites Based on Experimental Results

Some of the previous research showed good results and improvements in terms of partial discharge resistance. Thus, in order to know the polymer that reacts best with the nanofillers, we will look into five kinds of base polymers: epoxy, polyethylene, polyimide, polyamide, and polyethylene/natural rubber.

#### 2.5.1. Epoxy Nanocomposite

A lot of experiments were done to investigate the electrical properties of epoxy polymer nanocomposite from 2005 until 2011 [31–42], especially in PD resistance and voltage endurance of the composites after electrical stress. The epoxy resins were mixed with small amounts of nanolayered silicate, nanosilica, nanotitania, and nano alumina. Most of them demonstrated that the addition of the nanoparticles could greatly enhance the properties of the epoxy despite using the epoxy alone, based on the following results.

(i) A comparison of the dispersion erosion depth after 480 hours of voltage application results in reduction to 146 *µ*m for the base specimen, 57 *µ*m for the Nanopox specimen (prepared by dispersing nanosilica in epoxy resin and curing the formulated mixture) and 23 *µ*m for the Aerosil specimen (prepared by directly curing a mixture of epoxy and nanosilica) [37].
(ii) The erosion depth of epoxy/silicon carbide (SiC) specimens decreases with the increase of nanofiller content from 0 to 5 wt% [32].
(iii) The erosion depth of epoxy alumina nanocomposites due to PD decreases with increasing nanofiller content (3, 5, and 7 wt%) [35].
(iv) Discharge resistance increases with the increase of nanofiller concentration on the epoxy alumina nanocomposites from 0.1 wt% to 15 wt% [38]. In contrast, addition of alumina microcomposites gives inferior results.
(v) Nanocomposites take the longest breakdown time, which is 307 min, compared to neat epoxy (186 min), microcomposite (94 min), and nano-micro-composite (275 min) [39].
(vi) An increment of lifetime was observed on the nanocomposite material of nanosilicate filled epoxy resins and a higher shape of Weibull distribution in an internal discharge investigation, which means that the material becomes more homogeneous [26].

From these results, it was proven that by adding a low wt% of nanofiller concentration to the epoxy resins, the PD characteristic is remarkably improved. This is most likely due to the strong bonding between nanoparticles and the epoxy at the interfacial region, which causes the polymer material to hold on to the nanoparticles and resist degradation [40]. Addition of microfillers does not make any significant contribution to restraining PD erosion compared to nanosized fillers. However, microfillers can increase the thermal conductivity of epoxy composite as an advantage [39]. Due to such characteristics, some researchers considered combining the addition of microfillers and nanofillers in a composite to compensate for the drawbacks of the microfiller [31, 39].

Besides, there was also a study about the most compatible and best PD resistance of nanocomposite when added to epoxy resin. Kozako et al. [41] conducted an experiment on surface erosion due to PD on several kinds of epoxy nanocomposites, in which the specimens are listed as follows:

- epoxy + TiO_2_ 5 wt%, 15 nm size needle-like shape,
- epoxy + SiO_2_ 5 wt%, 12 nm size spherical shape,
- epoxy + SiO_2_ 5 wt%, 40 nm size spherical shape,
- epoxy + nano-scaled layered silicate (intercalated structure) 5 wt%.

Maintaining the same wt% of nanofiller, it was found that epoxy/SiO_2_ nanocomposites are more PD resistant than other nanocomposites. This could be related to the PD resistance of silica and the bonding strength between silica and epoxy matrices. Further, the smaller size of epoxy/SiO_2_ is superior in PD to that of a larger size, which could be related to its interfacial area.

In addition, this discovery was also strengthened by the results obtained by Tanaka et al. [33], who concluded that nanosilica performs better than nanolayered silicate and nanotitania based on their investigation of the nanoeffects on PD endurance of epoxy nanocomposite.

#### 2.5.2. Polyethylene Nanocomposites

Various types of polyethylene are used in investigating high voltage insulation as well as in applications [24, 28, 43–53]. Polyethylene is a thermoplastic polymer consisting of a long hydrocarbon chain. Most polyethylenes, such as low density polyethylene (LDPE), linear low density polyethylene (LLDPE), cross-linked polyethylene (XLPE), and high density polyethylene (HDPE), have a great resistance to electrical stress, thus making them useful as high voltage insulating material besides their primary use as packaging material, such as plastic. The characteristics of the electrical properties under investigation include electrical breakdown, partial discharge, and electrical treeing. The experimental works regarding this type of polymer are further explained in this section.

*(a) High Density Polyethylene (HDPE).* Not much has been published on the electrical properties of HDPE nanocomposites when used as insulating material. Shah et al. [50] reported that, generally, HDPE organoclay nanocomposite improves the electrical properties, including dielectric strength, volume resistivity, and surface resistivity. As the clay content was increased up to 5 wt%, the dielectric strength of the nanocomposite increased significantly. Besides, the clay particles in the compound are understood to perform as an obstacle for breakdown by electrical stress applied to it. Sami et al. [53] conducted experiments on the corona discharge of HDPE clay nanocomposite using the standard electrode configuration of the CIGRE method II. However no improvement of the resistance to corona discharge was obtained. This result is still under investigation.

*(b) Cross-Linked Polyethylene (XLPE).* The available results and data for this XLPE polymer with nanofiller are limited. Recently, in 2011, Tanaka et al. [49] reported evidence of the enhanced dielectric properties of XLPE nanocomposite especially toward the partial discharge resistance. The samples used in this experiment were based on standard commercial grade XLPE, to have more impact on improving the current insulation used for power extruded cables. Two methods of PD resistance evaluation were conducted in this investigation: the first by using a rod-to-plane electrode and the second similar to the IEC (b) electrode. The first method showed PD endurance that was significantly improved for the filled XLPE (with SiO_2_ nanofillers) compared to unfilled XLPE (without SiO_2_ nanofiller). The improvement was for filled XLPE with surface-treated filler. On the other hand, with the second method, which used an electrode similar to the IEC (b) electrode to test the three heat-treated samples (unfilled, filled SiO_2_ without and with surface-treated filler), no apparent improvement was made by the nanofillers. It was generally speculated that this is due to the effect of the filler treatment of the samples.

Hence, data analysis and tests by the second method should be further investigated to achieve satisfactory results. Overall, the nanofiller SiO_2_ (5%) significantly improved the PD resistance as it had modified the sample surface of XLPE-SiO_2_ nanocomposite. 

**Figure 5:**
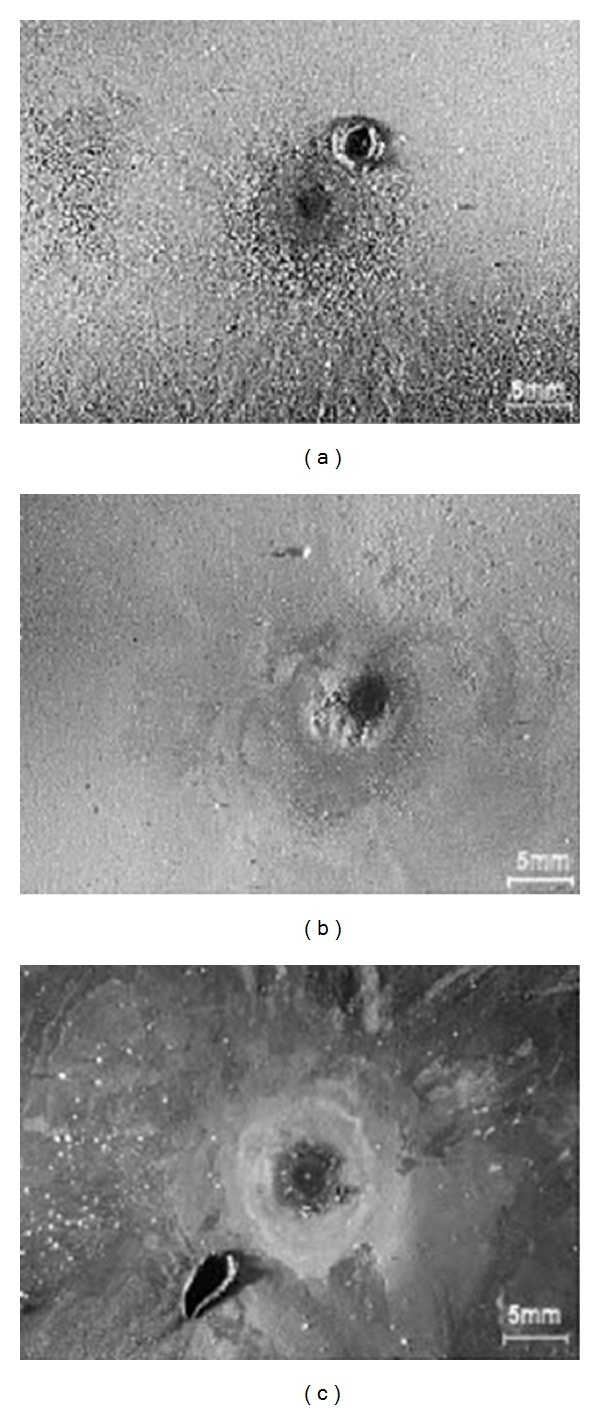
Images of the eroded surface of the specimens: (a) LDPE; (b) LDPE + MMT 5%; (c) LDPE + Si 5% [24].

*(c) Low-Density Polyethylene (LDPE).* LDPE is one of the most common types of polyethylene that is utilized as insulating material for investigation among researchers [24, 53]. For instance, Guastavino et al. [24] conducted a study on the behaviour of LDPE nanocomposite toward surface partial discharge. The samples used for this experiment were unfilled LDPE, LDPE + Si (5 wt%), and LDPE + MMT (5 wt%). The method adopted in this experiment used a sphere-plane electrode configuration and the test was carried out by applying alternating sinusoidal voltage having a frequency of 50 Hz and 7,500 V amplitude. The lifetime of each specimen was collected and compared. As expected, LDPE without filler has the lowest average lifetime compared to the filled LDPEs. Besides, it was observed that both LDPEs with nanofiller have smoother surfaces than unfilled LDPE, which had deeper erosion. Images of the eroded area on the tested specimens taken using an optical microscope are presented in Figure 5.

*(d) Linear Low-Density Polyethylene (LLDPE).* LLDPE also has limited literature and data on PD characteristics; hence, it was a challenge to the researcher to collect information about the performance towards the PD resistance. Makmud et al. [44, 45] conducted an experiment on LLDPE nanocomposite blended with natural rubber toward the PD performance, characteristics, and tensile properties. This proved that the total PD numbers decrease with the increase of the wt% of the nanofiller. Even though this experiment used natural rubber as part of the composition, it can be assumed that this polymer itself had its own role to restructure and recombine with nanocomposite for this experiment. From this point of view, the additional natural rubber in this experiment also provided a good path for future research in expanding the development of insulation instead of using only the polymer base with nanofiller.

#### 2.5.3. Polyimide Nanocomposite

Polyimide is used as the main insulating material in low voltage motors due to its excellent characteristic as organic dielectric. However, PD often occurs as a result of the high frequency square wave pulse in its operation. Due to this condition, Peihong et al. [54] were attracted to study the performance of polyimide nanoinorganic oxides composites as the insulating material in motors by studying the PD/corona mechanism. Samples of modified film and original film of polyimide nanoinorganic oxides composites with different components and contents were prepared. The test result showed that the modified film has better corona resistance than the original film, with the best compound of modified polyimide + 8% SiO_2_, which means the PD resistance was stronger for nanocomposites than for pure polyimide.

#### 2.5.4. Polyamide Nanocomposite

Kozako et al. [55] conducted an investigation on the properties of polyamide-6 nanocomposite as an insulating material because of its present commercial availability. They carried out experiments on four kinds of material, which are polyamide-6 without nanofillers and with 2 wt%, 4 wt%, and 5 wt% addition of nanofiller. Their PD resistance was examined using the IEC (b) electrode system and the surface roughness from scanning electron micrography (SEM) of each specimen was analyzed. It was found that the PD current property is almost identical for each type of specimen, where a small addition of nanofiller of 2 wt% does not significantly change the property of PD resistance. From the results, they concluded that polyamide nanocomposites exhibit much stronger PD resistance than pure polyamide. Meanwhile, from the SEM image observation, it seems that surface erosion due to PD was 5 times shallower for polyamide nanocomposite than for pure polyamide under certain conditions.

Fuse et al. in 2004 [56] had done the same investigation utilizing an IEC (b) electrode system with the preparation of three kinds of polyamide-6 nanocomposites sample, that is, addition of 2 wt%, 4 wt%, and 5 wt% layered silicate. Using an atomic force microscope (AFM), it was observed that the roughness of the samples' surface exposed to PD increases with an increase in the PD exposure period in all the samples. However, the increment is rapidly reduced when nanofiller is added to the samples. Hence, from the results, the authors agreed that the PD characteristic is superior in polyamide nanocomposites to that in conventional polyamide. Besides, the presence of layered silicate and strong ionic interaction at the interface between layered silicate and polyamide contributed to increasing the endurance against PD activity.

Guastavino et al. [57] investigated the short and medium/long-term performance of a nanofilled polyamide-imide enamel wire in the occurrence of PD. Using enamelled wire twisted pair specimens that followed the IEC 851-5 standard procedure, their behavior was compared with other two commercial wires based on electric strength tests and aging tests in the presence of PD. Amazingly, the outcome of the experiments proved that nanostructured organic-inorganic hybrid enamels can withstand the electrical stress due to pulsed voltage waveform together with PD activity better than the other two kinds of insulated wires for the low voltage electrical machines that are widely used, that is, polyamide-imide enamel and polyimide film.

#### 2.5.5. Polymer/Natural Rubber (NR) Nanocomposite

An experiment was conducted by Piah et al. [58] using the combination of LLDPE/NR without nanofiller. The results revealed that the sample of 80% LLDPE and 20% NR seems to be the best composition based on the least damaged and lowest degradation index. Some researchers have taken advantage of this finding to continue studying this combination with the addition of the nanofillers to increase performance in dielectric properties, and especially PD resistance. Makmud et al. [44, 45] studied this combination by using LLDPE/NR with nanofiller MMT and TiO_2_. Considering the PD resistance, the sample from LLDPE/NR with 4 wt% MMT seems to be the best composition due to the suppression of PD activities during the aging time.

## 3. Discussion

### 3.1. Applications of Nanocomposites in Power System

Nowadays, certain fields in the power system use nanocomposites to improve the material insulation. For example, in power delivery, the addition of ZnO in surge arresters results in excellent performance of that equipment, since electrical properties such as conductivity or permittivity are strongly field-dependent [21]. On the other hand, nanoparticles like TiO_2_ added to a polymer such as polyethylene have been investigated and studied for application in DC transmission. This kind of nanocomposite could mitigate the space charge accumulation that happens due to the large thermal gradient across the cable.

**Figure 6:**
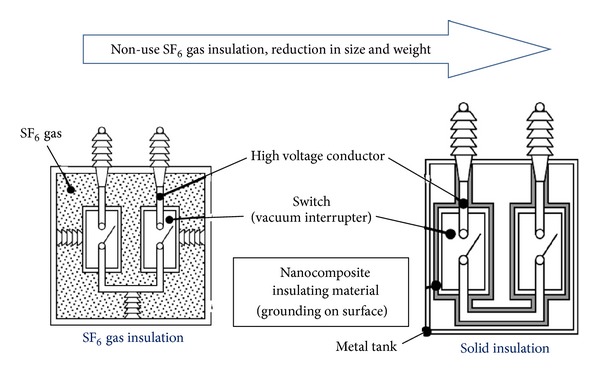
Example of nanocomposites in switchgear under development [25].

**Figure 7:**
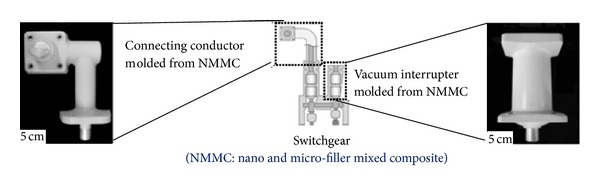
Development of switchgear components using nanocomposites [25].

In 2011, the latest technology development to apply nanocomposites as the insulating material in high voltage apparatus was heavy electrical apparatus such as switchgears, instead of using SF_6_ [25]. The development of solid insulation by utilizing nanocomposites that reduce the size and weight of heavy electrical apparatuses is as shown in Figure 6. The components of the switchgear have also been developed by using nanocomposites, as shown in Figure 7.

### 3.2. Processing Techniques Based on Previous Research

Many types of processing techniques or methods have been applied in order to prepare a sample of polymer nanocomposite, such as intercalation [43], ultrasonic agitation [34], direct mixing [34, 59, 60], fuming or super glue [22, 49], the sol-gel method [22, 60], organic modification [31], and solubilisation [31]. Besides, some researchers added a chemical coupling agent and curing agent into the polymer nanocomposite samples in their preparation to improve dispersion in the polymer [31, 49]. The nanofillers are compounded into the polymer using an intensive mixer, extrusion machine, or two-roll mill, to maximize dispersion and minimize aggregation of the nanofiller particles [49]. It is expected that well-dispersed filler in a polymer nanocomposite sample will give better electrical properties, such as partial discharge characteristics.

In this section, a few of these processing techniques will be highlighted as they are the most commonly employed by researchers in the high voltage insulating material field.

#### 3.2.1. Intercalation Method

**Figure 8:**
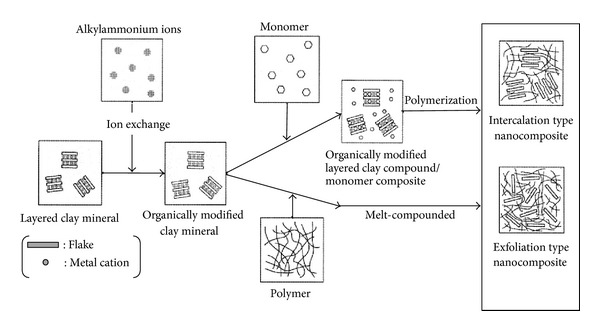
Intercalation and exfoliation process of nanocomposites [13].

The intercalation method consists of three submethods: polymer or prepolymer intercalation, in situ polymerization, and melts intercalation. Polymer or prepolymer intercalation from solution is a process of intercalating polymers or prepolymers between layers of inorganic layer substances based on a solvent system. The polymers or pre-polymers are in soluble form and the inorganic layer substances, such as silicate layers, that are swellable. The first process is swelling the layered silicate in solvent such as water, chloroform, or toluene, after which the solution is mixed with the soluble polymer, resulting in an intercalating and displacing process that occurs within the interlayer of silicate between the polymer chains and the solvent. At the end, a polymer with layered silicate nanocomposite is obtained. Secondly, the in situ polymerization method involves swelling the layered silicate in the monomer solution so that a polymer is formed between the intercalated sheets. The polymerization is later conducted by heating or an organic initiator or catalyst fixed through cation exchange inside the interlayer. Lastly, the melt intercalation method has great advantages compared to both polymer intercalation solution and in situ intercalation polymerization. This is for two reasons: first, it is good for the environment because no organic solvents are involved and second is the compatibility with industrial processes such as extrusion and injection molding. The process for this method looks simple as it involves annealing, statically or maybe under shear, a mixture of the polymer and layered silicate above the softening point of the polymer. The process of intercalation and exfoliation is shown in Figure 8.

#### 3.2.2. Sol-Gel Method

This method is traditionally used for fabricating glass and ceramics. Tanaka et al. [13] explained that the sol-gel will start to react from the metal alkoxide, M (OR)_*n*_ and is supposed to be melted in water, alcohol, acid, or ammonia. That metal alkoxide is then hydrolyzed through reaction with water and produces metal hydroxide and alcohol as a result. The example is the formation of three-dimensional network structures of silica by the polymerization reaction followed by hydrolysis [13].

(1) $$\begin{array}{c} \text{Si}{\left(\text{O}{\text{C}}_{\text{2}}{\text{H}}_{5}\right)}_{4}+{\text{H}}_{2}\text{O}⟶{\left(\text{O}{\text{C}}_{2}{\text{H}}_{5}\right)}_{3}\text{Si–OH}+{\text{C}}_{2}{\text{H}}_{5}\text{OH}\\ \equiv \text{Si–OH}+\text{HO–Si}\equiv ⟶\equiv \text{Si–O–Si}\equiv +{\text{H}}_{2}\text{O}\\ \equiv \text{Si–OH}+{\left(\text{O}{\text{C}}_{2}{\text{H}}_{5}\right)}_{3}\text{Si–}⟶\;\equiv \text{Si–O–Si}\equiv +{\text{C}}_{2}{\text{H}}_{5}\text{OH} \end{array}$$

#### 3.2.3. Direct Dispersion Method

This method seems to be simple, as Tanaka et al. [13] said that the nanoparticles are chemically modified on their surfaces in order to increase the compatibility, then mixed and dispersed homogeneously with the polymers without agglomeration. Examples of this method include a nanoparticles paste of gold, polyamide-6 nanocomposite with silica nanoparticles surface-treated by amino butyric acid, and many more.

### 3.3. Measurement Technique Based on Previous Research

There are several techniques for measurement of the PD resistance or erosion as follows.

#### 3.3.1. CIGRE Method II System

**Figure 9:**
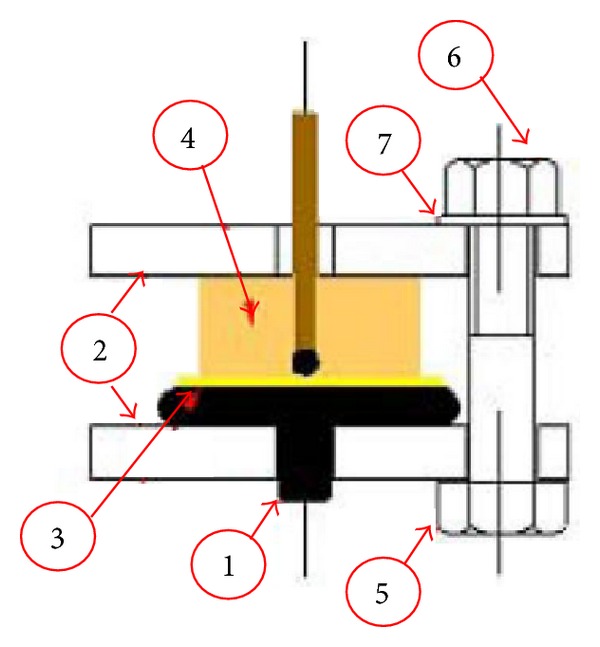
Test cell electrode system: (1) plane electrode; (2) acrylic plate; (3) kapton spacer; (4) Molded sphere electrode with specimen; (5) polycarbonate bolt; (6) polycarbonate nut; and (7) nylon washer [26].

Using this technique, the PD aging under surface discharge phenomena is observed and the sample can be evaluated for its long-term endurance as an insulating material. Casale et al. [26] investigated PD aging activity by using this method, applying 50 Hz sinusoidal voltage 30 kV_rms_ to the test cell which was dipped in mineral oil at room temperature. This method inspired other researchers to investigate PD characteristics on nanocomposite material [43–45, 47]. The cell electrode system is shown in Figure 9.

#### 3.3.2. IEC (b) Electrode System

**Figure 10:**
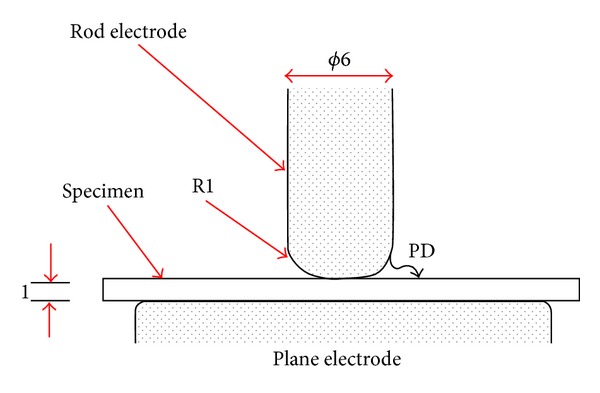
IEC (b) electrode configuration system consisting of a rod and a plane stainless-steel electrode [27].

This method is widely used for the measurement of PD degradation test. Kozako et al. [27], followed by other researchers [61], have conducted research using this method to investigate degradation due to surface discharge. The configuration of this method is shown in Figure 10. In the experiment, the diameter of the rod was 6mm with the end curvature of 1 mm radius. The authors applied alternating high voltage from 4 kV_rms_ up to 10 kV_rms_ with 50 Hz frequency to the specimens (slab shape) having dimensions of 60 mm × 60 mm × 1 mm. The period for applying the high voltage was about one hour up to 48 hours, due to the fact that the PDs were caused to occur at the edge of the rod electrode. This experiment was conducted in an acrylic cell with silica gel inside to maintain a humidity level similar to the ambient level.

#### 3.3.3. Rod-to-Plane Electrode System

**Figure 11:**
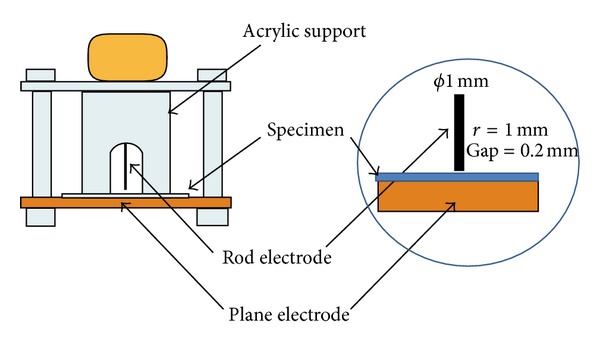
Rod-to-plane electrode system configuration with the 0.2 mm air gap [28].

This electrode system seems to be similar to the IEC (b) electrode, with the exception of about 0.2 mm air gap vertically implemented in this electrode system. Tanaka et al. [28] conducted an experiment using this method and their configuration is shown in Figure 11. A high voltage tungsten rod was placed vertically against a grounding plane electrode to form a pair of electrodes. Then a specimen of 1 mm in diameter was inserted between a pair of electrodes with an air gap of about 0.2 mm. The authors clearly stated that the specific gap was measured and set by using a metal thickness gauge, and epoxy glue was used to fix the tungsten rod in the center hole of the acrylic support. This experiment used a high-frequency high voltage source (Trek Model 610E HV Amplifier) with a Textronix AFG 320 function generator so that the PD degrades quickly for observation. The applied voltage was 4 kV at 720 Hz for a period up to 48 hours.

#### 3.3.4. Sphere Plane Electrode System

**Figure 12:**
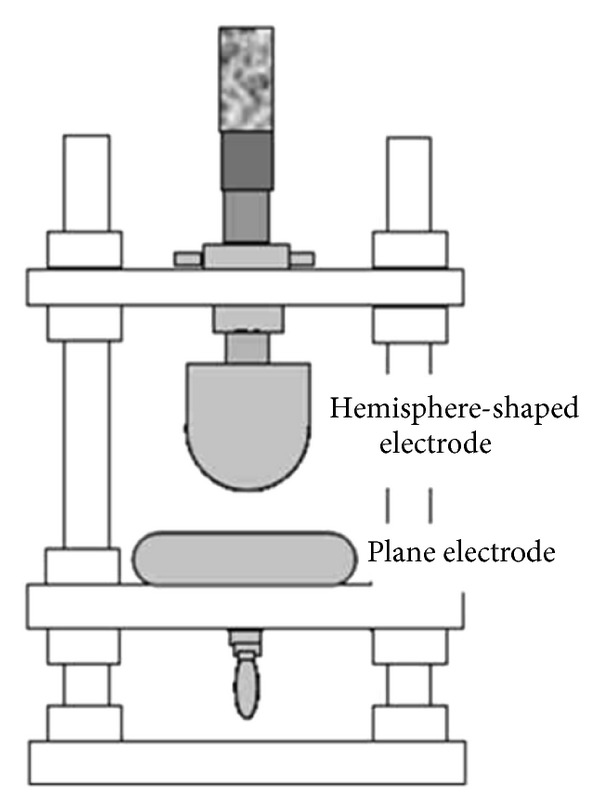
Sphere plane electrode system configuration [29].

This measurement system is not much used by researchers.  Figure 12 shows the configuration and specimen setup that was conducted by Higashiyama et al. [29] for investigating the breakdown voltages and partial discharge phenomena defects simultaneously. The 60 Hz frequency alternating voltage signal was supplied by a functional generator to an amplifier and then fed to the high voltage transformer.

### 3.4. Analysis Technique Based on Previous Research

Several techniques for the PD characteristic analysis have been used by researchers, which are stochastic, pulse shape and pulse sequence, and Weibull distribution analysis.

#### 3.4.1. Stochastic Analysis

The PD patterns can be derived analytically by using this technique. It generally evaluates the charge transferred during PD activities and measures the time or alternating current phase of the PD occurrences. Those discharges and the phase angle are of great importance for the analysis of phase-resolved partial discharge (PRPD). One of the papers published by Altenburger et al. [62] has an interesting approach towards the theories of PD. Though the authors are restricted to the discharge patterns of voids in solid insulation (epoxy resin), the concept seems to be similar and could be implemented with other kinds of discharges as well. The development of PD analysis via stochastic analysis contributes to the estimation of the physical discharge parameters of PD, especially the first electron availability and the charge removal upon discharge process.

#### 3.4.2. Pulse Shape and Pulse Sequence Analysis

**Figure 13:**
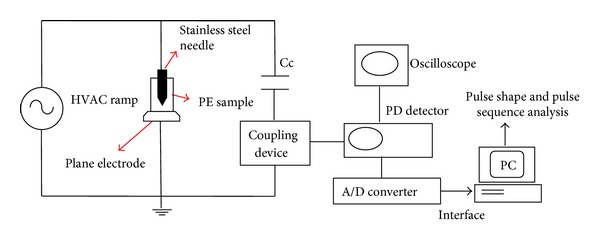
Simplified schematic diagram for PD measurement [30].

**Figure 14:**
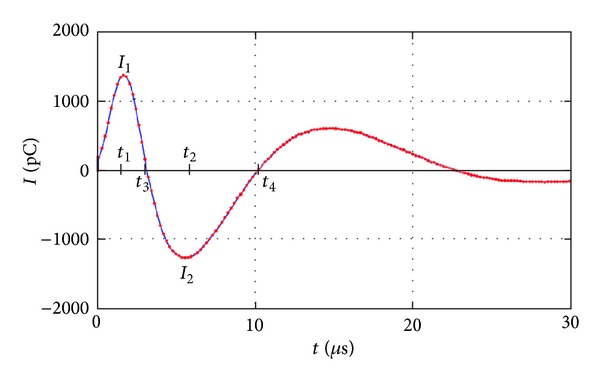
Typical PD and parameters for the pulse shape and pulse sequence analysis [30].

The second approach reviewed is pulse shape and pulse sequence analysis. Patsch et al. [30, 63] implemented this approach in their analysis for identifying the PD characteristics.  Figure 13 shows the simplified schematic diagram for PD measurement and pulse shape and pulse sequence analysis. In this experiment, the coupling device that was set up in series with the coupling capacitor, Cc, sensed the apparent PD signal and then the PD detector detected the PD magnitude and other parameters. The band pass filter used was within the range of 40–400 kHz for capturing the PD signal and noise discrimination. Then the analog signal was converted to a digital signal by an A/D converter and a PC captured and stored the signal for evaluation of the pulse shape and pulse sequence analysis. The typical PD signal and the parameters that were captured are as shown in Figure 14.

In this paper, every PD parameter was analyzed in detail and the results were clearly discussed to enable other researchers to understand the method. Lastly, the authors clearly stated that this kind of approach has proven to be a powerful tool for PD measurements compared with the conventional evaluation approach that focuses on the basis of phase angle occurrences only.

#### 3.4.3. Weibull Distribution Analysis

Another approach to analysis is by using the Weibull distribution. This approach is also widely used in the engineering field, especially to model the stochastic deterioration of partial discharge phenomena that occur in insulation [64]. One of the papers that review this Weibull distribution approach was published by Deshpande et al. [65], who highlighted that PD pattern recognition was of great importance in identifying PD characteristics or parameters. To execute the recognition, first we have to perform appropriate stochastic models that involve PD height in amplitude and phase distributions, which is also known as partial discharge height distribution (PDHD) analysis. Then the PD characteristics or statistical parameters can be found by proper interpretation of the resulting recognition of the PD source and degradation process. Different sources of discharge will produce different PDHD patterns. In the Weibull approach, a PDHD from a single PD signal has two parameters, which are *α* (scale parameters) and *β* (shape parameters). The Weibull functions as in (2) are the cumulative distribution and the probability density function for those two parameters [65].

Consider

(2) $$\begin{array}{c} F\left(q\right)=1-e\left[-{\left(\frac{q}{a}\right)}^{\beta }\right],\\ f\left(q\right)=\frac{\beta }{\alpha }{\left(\frac{q}{a}\right)}^{\beta -1}e\left[-{\left(\frac{q}{a}\right)}^{\beta }\right]. \end{array}$$

On the other hand, Schifani and Candela [66] found that the Weibull distribution gave different lines on a graph for a different number of PD sources, namely, for a single and multiple PD source.

### 3.5. Future Trends and Challenges

**Figure 15:**
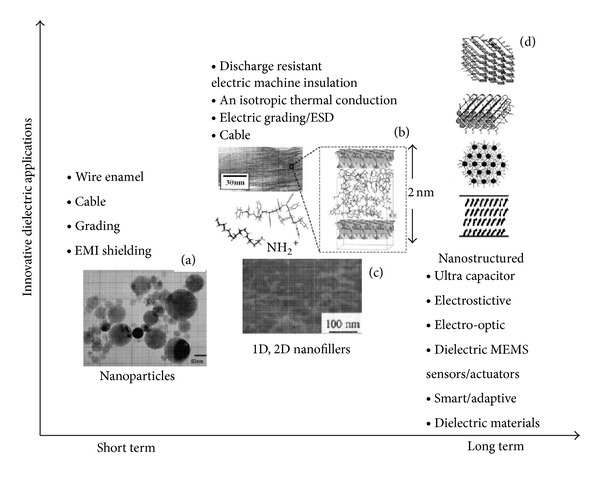
Recent and future development trends of nanocomposites over time [21].

As Cao et al. [21] said, it would be pleasing if we could tailor the use of nanocomposites to their dielectric properties such as controlled permittivity, conductivity, electric field, and frequency. Through this paper, it can be seen that the development of nanocomposites has been moving fast in recent years as it promises great improvements in the electrical properties of high voltage equipment, especially in terms of resistance to PD phenomena, as PD is among the major causes of serious faults in electrical systems. The research on filled systems through nanostructuration of dielectric material will gain extensive application. Briefly, the recent and future trends in nanocomposite development are as shown in Figure 15.

## 4. Concluding Remarks

In the early 21st century, nanocomposite materials have attracted great interest in the high voltage research field towards the improvement of insulation materials. For the long run, with the proof of such great experimental results, nanocomposites can be exploited widely as electrical insulating material, especially in the high voltage technology and engineering field. High voltage technology needs a material that is better in terms of physical strength, degradation performance, and high insulation integrity at an economical cost. With proper material, processing and design, this nanocomposite material can perform as the main factor in maximizing the lifespan of high voltage equipment and at the same time minimize maintenance costs.

## Figures and Tables

**Table 1:** Comparison between microcomposites and nanocomposites.

Properties	Microcomposite	Nanocomposite
Filler content	>50 wt%	<10 wt%
Filler size	10^−6^ m	10^−9^ m
Specific surface area of fillers	Small	Large
